# Supplementary material for: Unravelling the gender productivity gap in science: a meta-analytical review
Source: R Soc Open Sci. 2019 Jun 12;6(6):181566. doi: 10.1098/rsos.181566 (PMC6599789; doi:10.1098/rsos.181566)
Supplement: Supplementary information of meta-analyses [file rsos181566supp2.docx]

Supplementary material for

Unravelling the gender productivity gap in science: a meta-analytical review

Julia Astegiano^*^ , Esther Sebastián-González, Camila de Toledo Castanho

*correspondence to: [juastegiano@gmail.com](mailto:juastegiano@gmail.com)


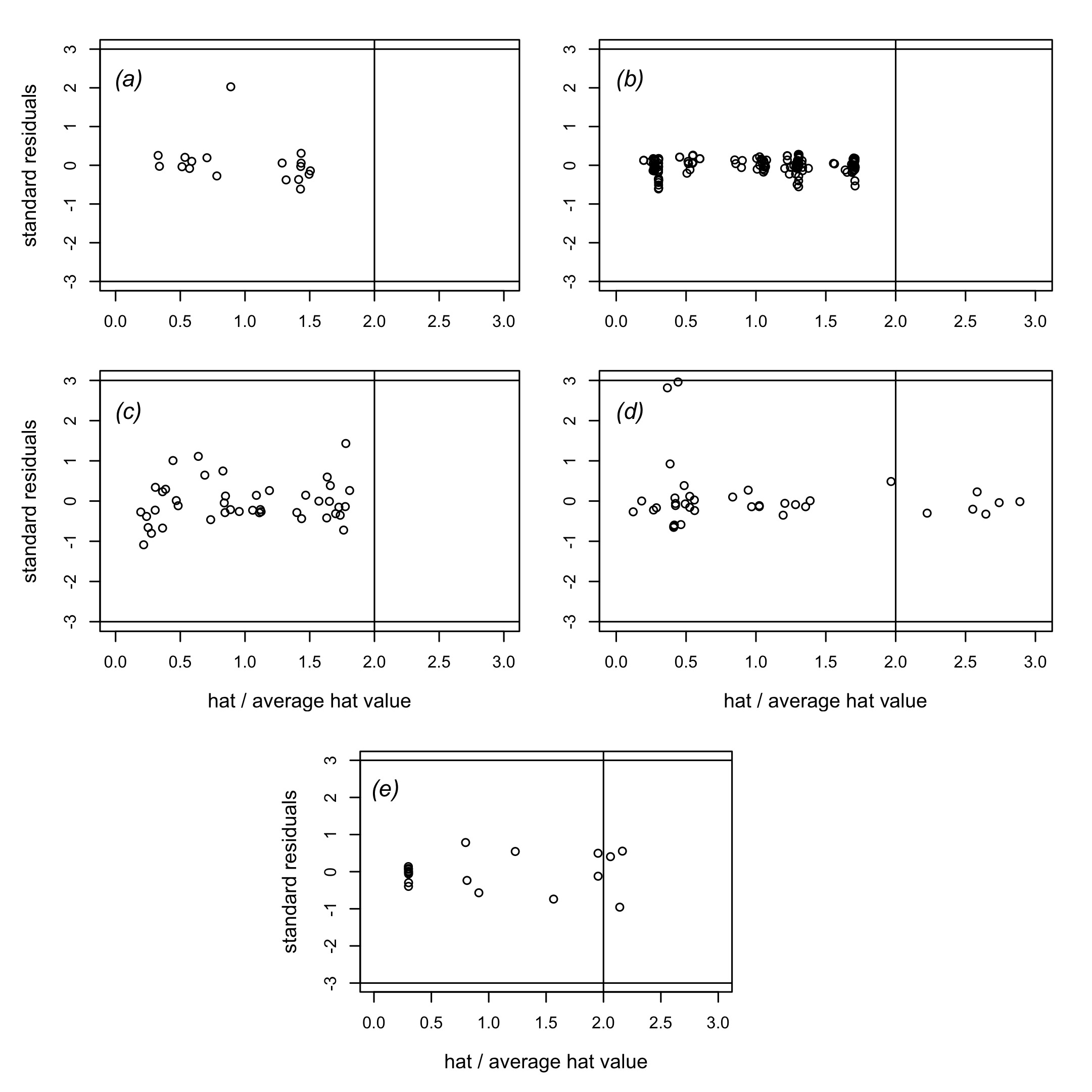


Figure S1. Sensitivity analyses results. Each panel represents the results corresponding to each question tested in this study. Influential outliers are effect sizes with hat values greater than two times the average hat value (vertical lines) and standardized residual values exceeding 3.0 (horizontal lines). *(a)* Question 1A: gender productivity gap (individual-based studies); *(b)* Question 1B: gender productivity gap (group-based studies); *(c)* Question 2: gender success rate; *(d)* Question 3: gender science impact; *(e)* Question 4: experimentally assessed gender-bias.

**Table S2** (*see Excel version*). Eligibility criteria. We include a list of the possible targets evaluated, study designs and data types for a study to be included in our meta-analysis

**Table S1.** Inclusion criteria checklist. We followed the following criteria to decide on the inclusion of a study in the meta-analyses and the inclusion of different outcomes from the same study.

| **Criteria** | **Decision** |
| --- | --- |
| STUDY  The study evaluates at least one of our study questions | Include |
| Response variable, study design and data type match our eligibility criteria list (Table S2) | Include |
| Response variable, study design and data type are not in our eligibility criteria list (Table S2) | Discuss if data should be included as new variable, design or data type |
| The study is an opinion article | Exclude |
| The study uses surveys to calculate productivity | Exclude |
| The study only compares wages among scientists | Exclude |
| The study did not provide the statistics needed | Ask data to authors |
| OUTCOMES |  |
| Study reports outcomes about different journals | Include each journal as an outcome only if the journals are from different research fields |
| Study reports outcomes from different sources | Include each source as an outcome only if the sources are independent |
| Study reports outcomes from several geographic regions | Include each geographic region as an observation |
| Study reports outcomes for several faculty levels (i.e. assistant, associate, full processor) | Include each level as an observation |
| Study reports outcomes for several editor levels (i.e. associate, subject, editor-in-chief) | Include each level as an observation |
| Study reports multiple aspects of the same data (e.g. CV hireability and quality) | Include only one of the proxies |
| Study reports outcomes about the first and last author of a set of articles | Include only outcomes about the first author |
| Study reports multiple outcomes across a given time period | Include most recent outcome. Include also the oldest outcome if there is a difference of at least a decade among them |
| The inclusion criteria are not clearly recognized in the study | Personally discuss study inclusion |
|  |  |

**Table S3.** Statistics measuring heterogeneity among effect sizes for each question tested in the meta-analyses. Q_total_ = statistic that estimates the variance among effect sizes. d.f. = Degrees of freedom. I^2^ = proportion of variance among effect sizes not attributable to sampling error but to true variance between studies.


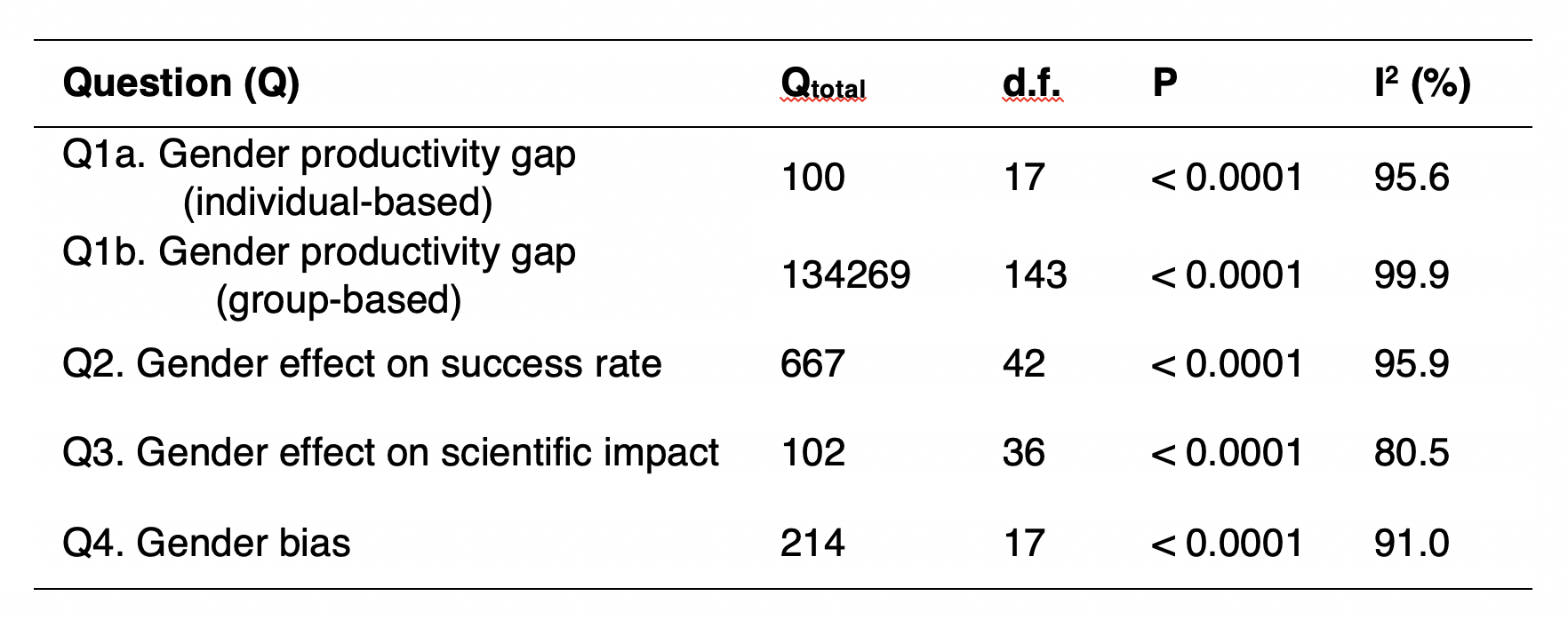


Table S4. Sample size (number of observations) and publication bias results for each question. Publication bias was accessed thought Egger’s regression.


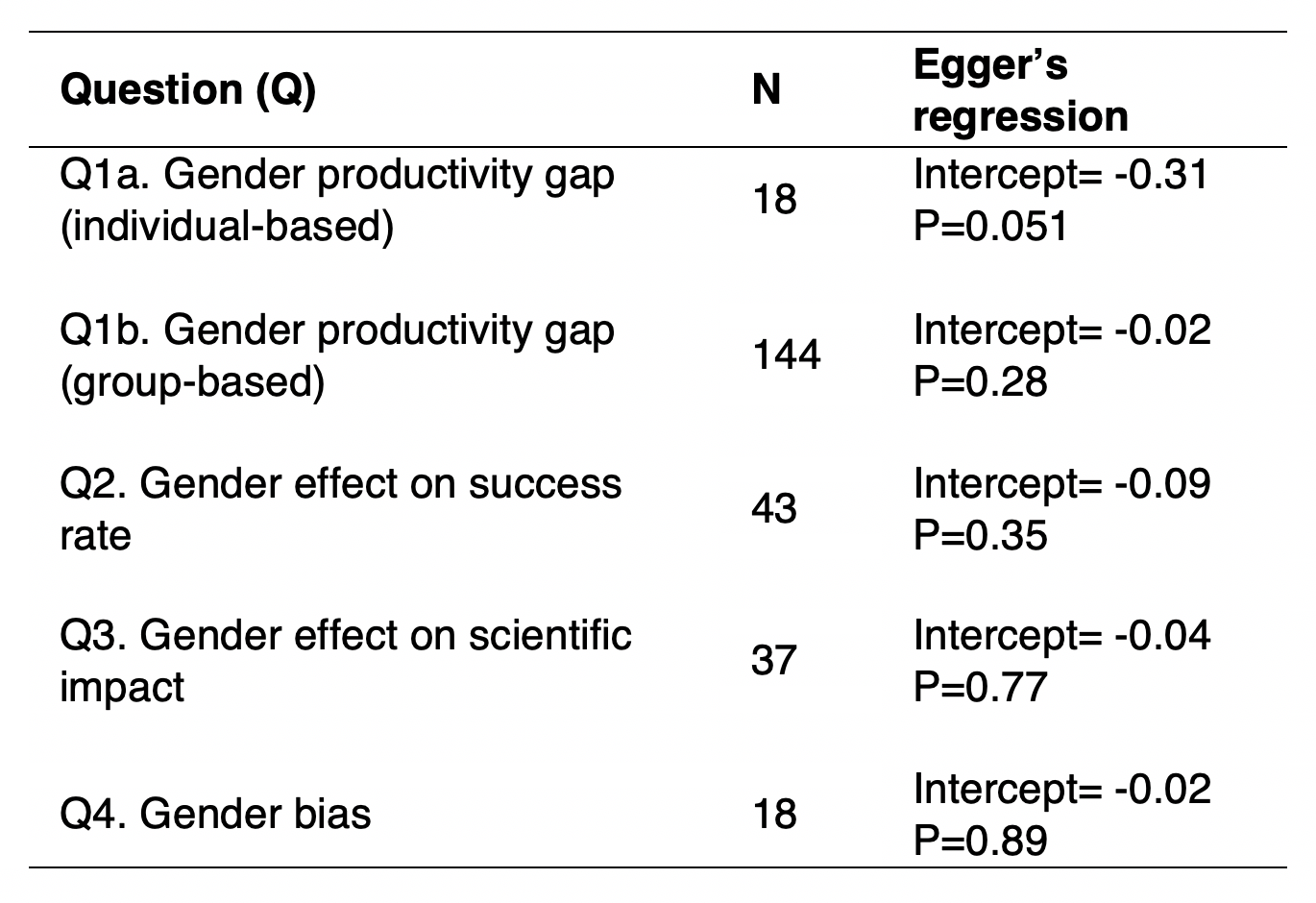


**Appendix S1.** Prisma checklist reporting for systematic review and meta-analyses.

**Datasets S1 to S7, combined into one Excel file:**

**Dataset S1.** References, study characteristics and effect sizes of the articles included in our meta-analysis that evaluated the gender productivity gap based on individual performance.

**Dataset S2.** References, study characteristics and effect sizes of the articles included in our meta-analysis that evaluated the gender productivity gap based on gender group performance.

**Dataset S3.** References, study characteristics and effect sizes of the articles included in our meta-analysis that evaluated the effect of gender on success rates.

**Dataset S4.** References, study characteristics, information about self-citation inclusion and effect sizes of the articles included in our meta-analysis that evaluated the effect of gender on science impact.

**Dataset S5.** References, study characteristics and effect sizes of the articles included in our meta-analysis that evaluated gender-bias based on experimental studies.

**Dataset S6.** Metadata of the datasets S1-S5.

**Dataset S7.** Complete references of the articles included in our meta-analyses.
