# Supplementary material for: Unravelling the gender productivity gap in science: a meta-analytical review
Source: R Soc Open Sci. 2019 Jun 12;6(6):181566. doi: 10.1098/rsos.181566 (PMC6599789; doi:10.1098/rsos.181566)
Supplement: Analyses code Q1a.html [file rsos181566supp7.html]

Unravelling the gender productivity gap in science


# Unravelling the gender productivity gap in science

### Question 1a - Gender productivity gap (individual-based)

#### Camila de Toledo Castanho

#### May 13, 2019

# Installing required packages

```
install.packages (c("metafor", "rmarkdown", "knitr")
```

# Loading required packages

```
library (metafor)
```

```
## Loading required package: Matrix
```

```
## Loading 'metafor' package (version 2.0-0). For an overview 
## and introduction to the package please type: help(metafor).
```

```
library(knitr)
library(rmarkdown)
```

# Loading data

```
Q1a<-read.table("Q1a.txt", header=TRUE, sep="\t")
str(Q1a)
```

```
## 'data.frame':    18 obs. of  12 variables:
##  $ ID_article      : int  68 69 69 218 239 395 396 435 441 486 ...
##  $ ID_observation  : int  1 2 3 4 5 6 7 8 9 10 ...
##  $ Reference       : Factor w/ 14 levels "Addessi et al. 2012",..: 6 1 1 11 2 12 10 8 7 13 ...
##  $ Time            : Factor w/ 3 levels "20th","21st",..: 3 2 2 3 3 1 1 NA NA 2 ...
##  $ Research_field  : Factor w/ 14 levels "Behavioral and educational sciences ",..: 3 9 9 4 7 11 8 2 13 5 ...
##  $ Research_field.2: Factor w/ 4 levels "Biological science",..: 1 1 1 1 1 3 3 1 4 4 ...
##  $ N_men           : int  233 72 88 129 305 2276 6230 262 255 71 ...
##  $ N_wom           : int  183 98 61 39 136 731 2937 138 162 27 ...
##  $ Mean_men        : num  3.02 1.25 1.71 28.26 12.4 ...
##  $ Mean_wom        : num  2.18 1.11 1.22 20.23 9.1 ...
##  $ SD_men          : num  1.29 1.19 1.03 18.13 9.35 ...
##  $ SD_wom          : num  0.95 0.79 0.13 11.27 6.25 ...
```

# Calculating effect sizes (yi) and variances (vi)

```
Q1a<-escalc(measure="SMD", m1i=Mean_men, m2i=Mean_wom, sd1i=SD_men, sd2i=SD_wom, n1i=N_men, n2i=N_wom, data=Q1a, vtype="UB")
Q1a
```

```
##    ID_article ID_observation                           Reference Time
## 1          68              1                  Krista et al. 2012  Mix
## 2          69              2                 Addessi et al. 2012 21st
## 3          69              3                 Addessi et al. 2012 21st
## 4         218              4                 Symonds et al. 2006  Mix
## 5         239              5                Braisher et al. 2005  Mix
## 6         395              6                  Xie & Shauman 1998 20th
## 7         396              7                     Sax et al. 2002 20th
## 8         435              8                    Long et al. 1993 <NA>
## 9         441              9                         Leahey 2006 <NA>
## 10        486             10 van den Besselaar & Sandstrom  2016 21st
## 11        486             11  van den Besselaar & Sandstrom 2016 21st
## 12        486             12  van den Besselaar & Sandstrom 2016 21st
## 13        625             13               Henderson et al. 2014 21st
## 14        781             14                 Mueller et al. 2017 <NA>
## 15        781             15                 Mueller et al. 2017 <NA>
## 16        781             16                 Mueller et al. 2017 <NA>
## 17        854             17                Frandsen et al. 2015  Mix
## 18       1001             18                  Cikara et al. 2012 21st
##                                          Research_field   Research_field.2
## 1                                               Biology Biological science
## 2                                           Primatology Biological science
## 3                                           Primatology Biological science
## 4                                 Ecology and Evolution Biological science
## 5                                          Life science Biological science
## 6                                               Several                Mix
## 7  Not informed, but we supposes all universities areas                Mix
## 8                                          Biochemistry Biological science
## 9                             Sociology and linguistics     Social science
## 10                                            Economics     Social science
## 11                 Behavioral and educational sciences      Social science
## 12                                          Psychology      Social science
## 13                                               Health             Health
## 14                                              Surgery             Health
## 15                                              Surgery             Health
## 16                                              Surgery             Health
## 17                                              Several                Mix
## 18                                     Social Psicology     Social science
##    N_men N_wom Mean_men Mean_wom  SD_men SD_wom      yi     vi
## 1    233   183     3.02     2.18    1.29   0.95  0.7272 0.0104
## 2     72    98     1.25     1.11    1.19   0.79  0.1423 0.0242
## 3     88    61     1.71     1.22    1.03   0.13  0.6119 0.0290
## 4    129    39    28.26    20.23   18.13  11.27  0.4756 0.0341
## 5    305   136    12.40     9.10    9.35   6.25  0.3868 0.0108
## 6   2276   731     4.02     3.05    5.45   4.46  0.1855 0.0018
## 7   6230  2937     2.79     2.47    1.20   1.09  0.2744 0.0005
## 8    262   138     1.41     1.61    0.98   1.08 -0.1966 0.0111
## 9    255   162    14.60     9.10   13.30   8.20  0.4736 0.0104
## 10    71    27     8.82     5.81    5.13   3.77  0.6222 0.0532
## 11    17    28    23.24    16.86   11.31  18.25  0.3912 0.0964
## 12    40    50    25.60    16.10   19.23  17.19  0.5197 0.0466
## 13   335    98   327.00   263.00 1373.71 785.05  0.0505 0.0132
## 14    51    23    23.47    16.56   22.58  14.21  0.3353 0.0639
## 15    45    10    45.69    25.50   32.07  11.85  0.6718 0.1266
## 16    59    18    85.78    61.83   66.60  44.85  0.3803 0.0735
## 17    63    78     0.88     0.77    2.17   3.11  0.0401 0.0287
## 18    30    23    28.20    15.50    6.30   2.90  2.4446 0.1375
```

# Hierarchical mixed effect meta-analysis

```
m.Q1a<-rma.mv(yi,vi, random=~1|ID_article/ID_observation,data=Q1a)
m.Q1a
```

```
## 
## Multivariate Meta-Analysis Model (k = 18; method: REML)
## 
## Variance Components: 
## 
##             estim    sqrt  nlvls  fixed                     factor
## sigma^2.1  0.1767  0.4203     13     no                 ID_article
## sigma^2.2  0.0210  0.1450     18     no  ID_article/ID_observation
## 
## Test for Heterogeneity: 
## Q(df = 17) = 100.0047, p-val < .0001
## 
## Model Results:
## 
## estimate      se    zval    pval   ci.lb   ci.ub    
##   0.4177  0.1287  3.2449  0.0012  0.1654  0.6700  **
## 
## ---
## Signif. codes:  0 '***' 0.001 '**' 0.01 '*' 0.05 '.' 0.1 ' ' 1
```

```
forest (m.Q1a, slab=Q1a$Reference, xlim=c(-5,6), cex=0.8, xlab = "Hedges'd", mlab = "Overall effect (18)")
text(-5,20, "Author(s) and Year", pos=4, font=2, cex=1)
text(6,20, "Hedges'd [95% CI]", pos=2, font=2, cex=1)
```

# Heterogeneity

Heterogeneity I^2 for hierarchical models is not provided by metafor. We calculate total heterogeneity using the formulas provided by Nakagawa & Santos 2012. 1) Calculate sampling variance of the dataset (we use precision of effect size); 2) Use the variance components of the model associated with random factors (those summarized in the sigma2 structure components).

## Sampling variance of the dataset

```
Q1a$wi <- 1/Q1a$vi 
sv.mQ1a <- sum(Q1a$wi*(length(Q1a$wi)-1))/(sum(Q1a$wi)^2-sum(Q1a$wi^2))
sv.mQ1a
```

```
## [1] 0.008995005
```

## Total heterogeneity

```
I2.total = (m.Q1a$sigma2[1]+m.Q1a$sigma2[2])/(m.Q1a$sigma2[1]+m.Q1a$sigma2[2] + sv.mQ1a) * 100
I2.total
```

```
## [1] 95.64836
```

# Publication bias

## Egger’s regression

Egger’s regression using the meta-analytic residuals as the response variable and the precision as the moderator, as proposed by Nakagawa & Santos 2012 for hierarchical models. If the intercept of Egger’s regression is significantly different from zero, there is evidence of publication bias.

```
egger.Q1a<-lm(residuals.rma(m.Q1a)~Q1a$vi)
summary(egger.Q1a)
```

```
## 
## Call:
## lm(formula = residuals.rma(m.Q1a) ~ Q1a$vi)
## 
## Residuals:
##      Min       1Q   Median       3Q      Max 
## -0.54079 -0.31719  0.03689  0.18078  1.16122 
## 
## Coefficients:
##             Estimate Std. Error t value Pr(>|t|)   
## (Intercept)  -0.3102     0.1473  -2.106  0.05133 . 
## Q1a$vi        8.5531     2.4945   3.429  0.00344 **
## ---
## Signif. codes:  0 '***' 0.001 '**' 0.01 '*' 0.05 '.' 0.1 ' ' 1
## 
## Residual standard error: 0.4296 on 16 degrees of freedom
## Multiple R-squared:  0.4236, Adjusted R-squared:  0.3875 
## F-statistic: 11.76 on 1 and 16 DF,  p-value: 0.003444
```

## Sensitivity analysis

If residual standard >3 AND hatvalue >2 times the average of hatvalues, run analysis with those cases deleted to test for sensitivity (from Habeck & Schultz 2015).

```
rs.Q1a.me<-rstandard (m.Q1a)
hat.Q1a.me<-hatvalues(m.Q1a)/mean(hatvalues(m.Q1a))
plot(hat.Q1a.me, rs.Q1a.me$resid, xlab="hat / average hat value", ylab= "standard residuals",xlim=c(0,2.5), ylim=c(-3,3), cex.lab=1.2)
abline (h=-3)
abline (h=3)
abline (v=(2))
```
