# Supplementary material for: Unravelling the gender productivity gap in science: a meta-analytical review
Source: R Soc Open Sci. 2019 Jun 12;6(6):181566. doi: 10.1098/rsos.181566 (PMC6599789; doi:10.1098/rsos.181566)
Supplement: Analyses code Q1b.html [file rsos181566supp10.html]

Unravelling the gender productivity gap in science


# Unravelling the gender productivity gap in science

### Question 1b - Gender productivity gap (group-based)

#### Camila de Toledo Castanho

#### March 21, 2019

# Installing required packages

```
install.packages (c("metafor", "rmarkdown", "knitr")
```

# Loading required packages

```
library(knitr)
library(rmarkdown)
library (metafor)
```

```
## Loading required package: Matrix
```

```
## Loading 'metafor' package (version 2.0-0). For an overview 
## and introduction to the package please type: help(metafor).
```

# Loading data

```
Q1b<-read.table("Q1b.txt", header=TRUE, sep="\t")
str(Q1b)
```

```
## 'data.frame':    144 obs. of  11 variables:
##  $ ID_Article        : int  35 35 41 84 84 110 134 140 140 144 ...
##  $ ID_observation    : int  1 2 3 4 5 6 7 8 9 10 ...
##  $ Reference         : Factor w/ 63 levels "Aki et al. 2015",..: 36 36 28 52 52 47 42 22 22 46 ...
##  $ Time              : Factor w/ 3 levels "20th","21st",..: 1 2 3 1 2 2 2 2 2 2 ...
##  $ Research_field    : Factor w/ 5 levels "Biological science",..: 2 2 4 2 2 1 2 2 2 2 ...
##  $ Productivity_proxy: Factor w/ 5 levels "evaluation comitees",..: 5 5 5 5 5 2 1 5 1 5 ...
##  $ men_perc          : num  94.8 70.8 17.2 20.9 15.1 ...
##  $ wom_perc          : num  5.2 29.2 82.8 79.1 84.9 ...
##  $ N_total           : int  669 930 8649 517 1088 361 372 2507 21 53 ...
##  $ N_men             : int  634 658 1488 108 164 265 323 1855 20 40 ...
##  $ N_wom             : int  35 272 7161 409 924 96 49 652 1 13 ...
```

# Calculating effect sizes (yi) and variances (vi)

```
Q1b<-escalc(measure="PR", xi=N_men, ni=N_total, data=Q1b)
head (Q1b)
```

```
##   ID_Article ID_observation              Reference Time     Research_field
## 1         35              1    Mansour et al. 2012 20th             Health
## 2         35              2    Mansour et al. 2012 21st             Health
## 3         41              3 Kretschmer et al. 2012  Mix     Social science
## 4         84              4    Shields et al. 2011 20th             Health
## 5         84              5    Shields et al. 2011 21st             Health
## 6        110              6     Rani & Luthra 2011 21st Biological science
##   Productivity_proxy men_perc wom_perc N_total N_men N_wom     yi     vi
## 1       publications    94.80     5.20     669   634    35 0.9477 0.0001
## 2       publications    70.80    29.20     930   658   272 0.7075 0.0002
## 3       publications    17.20    82.80    8649  1488  7161 0.1720 0.0000
## 4       publications    20.89    79.11     517   108   409 0.2089 0.0003
## 5       publications    15.10    84.90    1088   164   924 0.1507 0.0001
## 6             grants    73.40    26.60     361   265    96 0.7341 0.0005
```

# Hierarchical mixed effect meta-analysis

```
m.Q1b<-rma.mv(yi, vi, random=~1|ID_Article/ID_observation,data=Q1b)
m.Q1b
```

```
## 
## Multivariate Meta-Analysis Model (k = 144; method: REML)
## 
## Variance Components: 
## 
##             estim    sqrt  nlvls  fixed                     factor
## sigma^2.1  0.0108  0.1039     62     no                 ID_Article
## sigma^2.2  0.0241  0.1554    144     no  ID_Article/ID_observation
## 
## Test for Heterogeneity: 
## Q(df = 143) = 134268.8531, p-val < .0001
## 
## Model Results:
## 
## estimate      se     zval    pval   ci.lb   ci.ub     
##   0.7057  0.0204  34.6388  <.0001  0.6658  0.7456  ***
## 
## ---
## Signif. codes:  0 '***' 0.001 '**' 0.01 '*' 0.05 '.' 0.1 ' ' 1
```

```
forest (m.Q1b,  slab=Q1b$Reference, ylim=c(-1, 148), xlab="Raw proportion", mlab="Overall effect (144)")
abline(v=0.5)
text(-1.4,150, "Author(s) and Year", pos=4, font=2, cex=0.8)
text(2.7,150, "Raw proportion [95% CI]", pos=2, font=2, cex=0.8)
```

# Heterogeneity

Heterogeneity I^2 for hierarchical models is not provided by metafor. We calculate total heterogeneity using the formulas provided by Nakagawa & Santos 2012. 1) Calculate sampling variance of the dataset (we use precision of effect size); 2) Use the variance components of the model associated with random factors (those summarized in the sigma2 structure components).

## Sampling variance of the dataset

```
## [1] 1.068752e-05
```

## Total heterogenity

```
I2.total = (m.Q1b$sigma2[1]+m.Q1b$sigma2[2])/(m.Q1b$sigma2[1]+m.Q1b$sigma2[2] + sv.mQ1b) * 100
I2.total
```

```
## [1] 99.96943
```

# Moderators

## Research field

### Significance of the moderator

This parameterization of the model is used to test the significance of the moderator.

```
tapply(Q1b$ID_observation, Q1b$Research_field, length)
```

```
## Biological science             Health                Mix 
##                 23                 74                  9 
##     Social science              TEMCP 
##                 14                 24
```

```
m.Q1b_area <- rma.mv(yi, vi, mods= ~ Research_field, random=~1|ID_Article/ID_observation, data=Q1b)
m.Q1b_area
```

```
## 
## Multivariate Meta-Analysis Model (k = 144; method: REML)
## 
## Variance Components: 
## 
##             estim    sqrt  nlvls  fixed                     factor
## sigma^2.1  0.0118  0.1086     62     no                 ID_Article
## sigma^2.2  0.0233  0.1525    144     no  ID_Article/ID_observation
## 
## Test for Residual Heterogeneity: 
## QE(df = 139) = 125041.7511, p-val < .0001
## 
## Test of Moderators (coefficient(s) 2:5): 
## QM(df = 4) = 6.1348, p-val = 0.1893
## 
## Model Results:
## 
##                               estimate      se     zval    pval    ci.lb
## intrcpt                         0.6730  0.0417  16.1455  <.0001   0.5913
## Research_fieldHealth            0.0385  0.0472   0.8152  0.4150  -0.0540
## Research_fieldMix               0.1271  0.0731   1.7384  0.0821  -0.0162
## Research_fieldSocial science   -0.0329  0.0630  -0.5217  0.6019  -0.1564
## Research_fieldTEMCP             0.0737  0.0551   1.3380  0.1809  -0.0343
##                                ci.ub     
## intrcpt                       0.7547  ***
## Research_fieldHealth          0.1309     
## Research_fieldMix             0.2705    .
## Research_fieldSocial science  0.0906     
## Research_fieldTEMCP           0.1817     
## 
## ---
## Signif. codes:  0 '***' 0.001 '**' 0.01 '*' 0.05 '.' 0.1 ' ' 1
```

### Estimation and significance of each level

This parameterization of the model is used to estimate the mean effect size of each level of the moderator and test which of them are different from 0.5.

```
m.Q1b_area <- rma.mv(yi, vi, mods= ~ Research_field-1, random=~1|ID_Article/ID_observation, data=Q1b)
m.Q1b_area
```

```
## 
## Multivariate Meta-Analysis Model (k = 144; method: REML)
## 
## Variance Components: 
## 
##             estim    sqrt  nlvls  fixed                     factor
## sigma^2.1  0.0118  0.1086     62     no                 ID_Article
## sigma^2.2  0.0233  0.1525    144     no  ID_Article/ID_observation
## 
## Test for Residual Heterogeneity: 
## QE(df = 139) = 125041.7511, p-val < .0001
## 
## Test of Moderators (coefficient(s) 1:5): 
## QM(df = 5) = 1178.8646, p-val < .0001
## 
## Model Results:
## 
##                                   estimate      se     zval    pval
## Research_fieldBiological science    0.6730  0.0417  16.1455  <.0001
## Research_fieldHealth                0.7114  0.0259  27.4888  <.0001
## Research_fieldMix                   0.8001  0.0627  12.7613  <.0001
## Research_fieldSocial science        0.6401  0.0506  12.6624  <.0001
## Research_fieldTEMCP                 0.7467  0.0440  16.9608  <.0001
##                                    ci.lb   ci.ub     
## Research_fieldBiological science  0.5913  0.7547  ***
## Research_fieldHealth              0.6607  0.7622  ***
## Research_fieldMix                 0.6772  0.9230  ***
## Research_fieldSocial science      0.5410  0.7392  ***
## Research_fieldTEMCP               0.6604  0.8330  ***
## 
## ---
## Signif. codes:  0 '***' 0.001 '**' 0.01 '*' 0.05 '.' 0.1 ' ' 1
```

### Change in the reference level

This parameterization is used to test if social science (as reference level) is significantly different from the other levels of the moderator.

```
m.Q1b_area <- rma.mv(yi, vi, mods= ~ relevel (factor(Research_field), ref="Social science"), random=~1|ID_Article/ID_observation, data=Q1b)
m.Q1b_area
```

```
## 
## Multivariate Meta-Analysis Model (k = 144; method: REML)
## 
## Variance Components: 
## 
##             estim    sqrt  nlvls  fixed                     factor
## sigma^2.1  0.0118  0.1086     62     no                 ID_Article
## sigma^2.2  0.0233  0.1525    144     no  ID_Article/ID_observation
## 
## Test for Residual Heterogeneity: 
## QE(df = 139) = 125041.7511, p-val < .0001
## 
## Test of Moderators (coefficient(s) 2:5): 
## QM(df = 4) = 6.1348, p-val = 0.1893
## 
## Model Results:
## 
##                                                                            estimate
## intrcpt                                                                      0.6401
## relevel(factor(Research_field), ref = "Social science")Biological science    0.0329
## relevel(factor(Research_field), ref = "Social science")Health                0.0713
## relevel(factor(Research_field), ref = "Social science")Mix                   0.1600
## relevel(factor(Research_field), ref = "Social science")TEMCP                 0.1066
##                                                                                se
## intrcpt                                                                    0.0506
## relevel(factor(Research_field), ref = "Social science")Biological science  0.0630
## relevel(factor(Research_field), ref = "Social science")Health              0.0548
## relevel(factor(Research_field), ref = "Social science")Mix                 0.0766
## relevel(factor(Research_field), ref = "Social science")TEMCP               0.0615
##                                                                               zval
## intrcpt                                                                    12.6624
## relevel(factor(Research_field), ref = "Social science")Biological science   0.5217
## relevel(factor(Research_field), ref = "Social science")Health               1.3011
## relevel(factor(Research_field), ref = "Social science")Mix                  2.0897
## relevel(factor(Research_field), ref = "Social science")TEMCP                1.7339
##                                                                              pval
## intrcpt                                                                    <.0001
## relevel(factor(Research_field), ref = "Social science")Biological science  0.6019
## relevel(factor(Research_field), ref = "Social science")Health              0.1932
## relevel(factor(Research_field), ref = "Social science")Mix                 0.0366
## relevel(factor(Research_field), ref = "Social science")TEMCP               0.0829
##                                                                              ci.lb
## intrcpt                                                                     0.5410
## relevel(factor(Research_field), ref = "Social science")Biological science  -0.0906
## relevel(factor(Research_field), ref = "Social science")Health              -0.0361
## relevel(factor(Research_field), ref = "Social science")Mix                  0.0099
## relevel(factor(Research_field), ref = "Social science")TEMCP               -0.0139
##                                                                             ci.ub
## intrcpt                                                                    0.7392
## relevel(factor(Research_field), ref = "Social science")Biological science  0.1564
## relevel(factor(Research_field), ref = "Social science")Health              0.1788
## relevel(factor(Research_field), ref = "Social science")Mix                 0.3101
## relevel(factor(Research_field), ref = "Social science")TEMCP               0.2270
##                                                                               
## intrcpt                                                                    ***
## relevel(factor(Research_field), ref = "Social science")Biological science     
## relevel(factor(Research_field), ref = "Social science")Health                 
## relevel(factor(Research_field), ref = "Social science")Mix                   *
## relevel(factor(Research_field), ref = "Social science")TEMCP                 .
## 
## ---
## Signif. codes:  0 '***' 0.001 '**' 0.01 '*' 0.05 '.' 0.1 ' ' 1
```

## Time

### Significance of the moderator

This parameterization of the model is used to test the significance of the moderator.

```
tapply(Q1b$Time, Q1b$Time, length)
```

```
## 20th 21st  Mix 
##   51   77   16
```

```
m.Q1b_ano<-rma.mv(yi, vi, mods= ~ Time, random=~1|ID_Article/ID_observation, data=Q1b)
m.Q1b_ano
```

```
## 
## Multivariate Meta-Analysis Model (k = 144; method: REML)
## 
## Variance Components: 
## 
##             estim    sqrt  nlvls  fixed                     factor
## sigma^2.1  0.0111  0.1051     62     no                 ID_Article
## sigma^2.2  0.0244  0.1563    144     no  ID_Article/ID_observation
## 
## Test for Residual Heterogeneity: 
## QE(df = 141) = 110084.6294, p-val < .0001
## 
## Test of Moderators (coefficient(s) 2:3): 
## QM(df = 2) = 0.1012, p-val = 0.9507
## 
## Model Results:
## 
##           estimate      se     zval    pval    ci.lb   ci.ub     
## intrcpt     0.7126  0.0301  23.6991  <.0001   0.6536  0.7715  ***
## Time21st   -0.0083  0.0317  -0.2618  0.7935  -0.0703  0.0538     
## TimeMix    -0.0154  0.0594  -0.2596  0.7952  -0.1319  0.1011     
## 
## ---
## Signif. codes:  0 '***' 0.001 '**' 0.01 '*' 0.05 '.' 0.1 ' ' 1
```

### Estimation and significance of each level

This parameterization of the model is used to estimate the mean effect size of each level of the moderator and test which of them are different from 0.5.

```
m.Q1b_ano<-rma.mv(yi, vi, mods= ~ Time-1, random=~1|ID_Article/ID_observation, data=Q1b)
m.Q1b_ano
```

```
## 
## Multivariate Meta-Analysis Model (k = 144; method: REML)
## 
## Variance Components: 
## 
##             estim    sqrt  nlvls  fixed                     factor
## sigma^2.1  0.0111  0.1051     62     no                 ID_Article
## sigma^2.2  0.0244  0.1563    144     no  ID_Article/ID_observation
## 
## Test for Residual Heterogeneity: 
## QE(df = 141) = 110084.6294, p-val < .0001
## 
## Test of Moderators (coefficient(s) 1:3): 
## QM(df = 3) = 1180.3329, p-val < .0001
## 
## Model Results:
## 
##           estimate      se     zval    pval   ci.lb   ci.ub     
## Time20th    0.7126  0.0301  23.6991  <.0001  0.6536  0.7715  ***
## Time21st    0.7043  0.0251  28.0560  <.0001  0.6551  0.7535  ***
## TimeMix     0.6971  0.0514  13.5629  <.0001  0.5964  0.7979  ***
## 
## ---
## Signif. codes:  0 '***' 0.001 '**' 0.01 '*' 0.05 '.' 0.1 ' ' 1
```

## Productivity proxy

### Significance of the moderator

This parameterization of the model is used to test the significance of the moderator.

```
tapply(Q1b$ID_observation, Q1b$Productivity_proxy, length)
```

```
## evaluation comitees              grants               other 
##                  24                  10                   4 
##             patents        publications 
##                   3                 103
```

```
m.Q1b_prod<-rma.mv(yi, vi, mods= ~ Productivity_proxy,random=~1|ID_Article/ID_observation, data=Q1b)
m.Q1b_prod
```

```
## 
## Multivariate Meta-Analysis Model (k = 144; method: REML)
## 
## Variance Components: 
## 
##             estim    sqrt  nlvls  fixed                     factor
## sigma^2.1  0.0099  0.0994     62     no                 ID_Article
## sigma^2.2  0.0233  0.1526    144     no  ID_Article/ID_observation
## 
## Test for Residual Heterogeneity: 
## QE(df = 139) = 111785.7800, p-val < .0001
## 
## Test of Moderators (coefficient(s) 2:5): 
## QM(df = 4) = 10.2959, p-val = 0.0357
## 
## Model Results:
## 
##                                 estimate      se     zval    pval    ci.lb
## intrcpt                           0.8047  0.0445  18.0964  <.0001   0.7175
## Productivity_proxygrants         -0.0607  0.0753  -0.8062  0.4201  -0.2082
## Productivity_proxyother          -0.0430  0.1099  -0.3914  0.6955  -0.2583
## Productivity_proxypatents         0.0397  0.1278   0.3106  0.7561  -0.2107
## Productivity_proxypublications   -0.1341  0.0473  -2.8351  0.0046  -0.2268
##                                   ci.ub     
## intrcpt                          0.8918  ***
## Productivity_proxygrants         0.0868     
## Productivity_proxyother          0.1723     
## Productivity_proxypatents        0.2901     
## Productivity_proxypublications  -0.0414   **
## 
## ---
## Signif. codes:  0 '***' 0.001 '**' 0.01 '*' 0.05 '.' 0.1 ' ' 1
```

### Estimation and significance of each level

This parameterization of the model is used to estimate the mean effect size of each level of the moderator and test which of them are different from 0.5.

```
m.Q1b_prod<-rma.mv(yi, vi, mods= ~ Productivity_proxy-1,random=~1|ID_Article/ID_observation, data=Q1b)
m.Q1b_prod
```

```
## 
## Multivariate Meta-Analysis Model (k = 144; method: REML)
## 
## Variance Components: 
## 
##             estim    sqrt  nlvls  fixed                     factor
## sigma^2.1  0.0099  0.0994     62     no                 ID_Article
## sigma^2.2  0.0233  0.1526    144     no  ID_Article/ID_observation
## 
## Test for Residual Heterogeneity: 
## QE(df = 139) = 111785.7800, p-val < .0001
## 
## Test of Moderators (coefficient(s) 1:5): 
## QM(df = 5) = 1284.8242, p-val < .0001
## 
## Model Results:
## 
##                                        estimate      se     zval    pval
## Productivity_proxyevaluation comitees    0.8047  0.0445  18.0964  <.0001
## Productivity_proxygrants                 0.7440  0.0607  12.2500  <.0001
## Productivity_proxyother                  0.7617  0.1005   7.5816  <.0001
## Productivity_proxypatents                0.8443  0.1198   7.0490  <.0001
## Productivity_proxypublications           0.6706  0.0232  28.9544  <.0001
##                                         ci.lb   ci.ub     
## Productivity_proxyevaluation comitees  0.7175  0.8918  ***
## Productivity_proxygrants               0.6249  0.8630  ***
## Productivity_proxyother                0.5648  0.9586  ***
## Productivity_proxypatents              0.6096  1.0791  ***
## Productivity_proxypublications         0.6252  0.7160  ***
## 
## ---
## Signif. codes:  0 '***' 0.001 '**' 0.01 '*' 0.05 '.' 0.1 ' ' 1
```

### Change in the reference level

This parameterization is used to test if published articles (as reference level) is significantly different from the other levels of the moderator.

```
m.Q1b_prod<-rma.mv(yi, vi, mods= ~ relevel (factor(Productivity_proxy), ref="publications"),random=~1|ID_Article/ID_observation, data=Q1b)
m.Q1b_prod
```

```
## 
## Multivariate Meta-Analysis Model (k = 144; method: REML)
## 
## Variance Components: 
## 
##             estim    sqrt  nlvls  fixed                     factor
## sigma^2.1  0.0099  0.0994     62     no                 ID_Article
## sigma^2.2  0.0233  0.1526    144     no  ID_Article/ID_observation
## 
## Test for Residual Heterogeneity: 
## QE(df = 139) = 111785.7800, p-val < .0001
## 
## Test of Moderators (coefficient(s) 2:5): 
## QM(df = 4) = 10.2959, p-val = 0.0357
## 
## Model Results:
## 
##                                                                               estimate
## intrcpt                                                                         0.6706
## relevel(factor(Productivity_proxy), ref = "publications")evaluation comitees    0.1341
## relevel(factor(Productivity_proxy), ref = "publications")grants                 0.0734
## relevel(factor(Productivity_proxy), ref = "publications")other                  0.0911
## relevel(factor(Productivity_proxy), ref = "publications")patents                0.1738
##                                                                                   se
## intrcpt                                                                       0.0232
## relevel(factor(Productivity_proxy), ref = "publications")evaluation comitees  0.0473
## relevel(factor(Productivity_proxy), ref = "publications")grants               0.0650
## relevel(factor(Productivity_proxy), ref = "publications")other                0.1031
## relevel(factor(Productivity_proxy), ref = "publications")patents              0.1220
##                                                                                  zval
## intrcpt                                                                       28.9544
## relevel(factor(Productivity_proxy), ref = "publications")evaluation comitees   2.8351
## relevel(factor(Productivity_proxy), ref = "publications")grants                1.1292
## relevel(factor(Productivity_proxy), ref = "publications")other                 0.8834
## relevel(factor(Productivity_proxy), ref = "publications")patents               1.4242
##                                                                                 pval
## intrcpt                                                                       <.0001
## relevel(factor(Productivity_proxy), ref = "publications")evaluation comitees  0.0046
## relevel(factor(Productivity_proxy), ref = "publications")grants               0.2588
## relevel(factor(Productivity_proxy), ref = "publications")other                0.3770
## relevel(factor(Productivity_proxy), ref = "publications")patents              0.1544
##                                                                                 ci.lb
## intrcpt                                                                        0.6252
## relevel(factor(Productivity_proxy), ref = "publications")evaluation comitees   0.0414
## relevel(factor(Productivity_proxy), ref = "publications")grants               -0.0540
## relevel(factor(Productivity_proxy), ref = "publications")other                -0.1110
## relevel(factor(Productivity_proxy), ref = "publications")patents              -0.0654
##                                                                                ci.ub
## intrcpt                                                                       0.7160
## relevel(factor(Productivity_proxy), ref = "publications")evaluation comitees  0.2268
## relevel(factor(Productivity_proxy), ref = "publications")grants               0.2008
## relevel(factor(Productivity_proxy), ref = "publications")other                0.2931
## relevel(factor(Productivity_proxy), ref = "publications")patents              0.4129
##                                                                                  
## intrcpt                                                                       ***
## relevel(factor(Productivity_proxy), ref = "publications")evaluation comitees   **
## relevel(factor(Productivity_proxy), ref = "publications")grants                  
## relevel(factor(Productivity_proxy), ref = "publications")other                   
## relevel(factor(Productivity_proxy), ref = "publications")patents                 
## 
## ---
## Signif. codes:  0 '***' 0.001 '**' 0.01 '*' 0.05 '.' 0.1 ' ' 1
```

# Publication bias

## Egger’s regression

Egger’s regression using the meta-analytic residuals as the response variable and the precision as the moderator, as proposed by Nakagawa & Santos 2012 for hierarchical models. If the intercept of Egger’s regression is significantly different from zero, there is evidence of publication bias.

```
egger.Q1b<-lm(residuals.rma(m.Q1b)~Q1b$vi)
summary(egger.Q1b)
```

```
## 
## Call:
## lm(formula = residuals.rma(m.Q1b) ~ Q1b$vi)
## 
## Residuals:
##      Min       1Q   Median       3Q      Max 
## -0.59305 -0.08909  0.01835  0.14153  0.29866 
## 
## Coefficients:
##             Estimate Std. Error t value Pr(>|t|)
## (Intercept) -0.01793    0.01663  -1.078    0.283
## Q1b$vi       3.58418    2.49714   1.435    0.153
## 
## Residual standard error: 0.19 on 142 degrees of freedom
## Multiple R-squared:  0.0143, Adjusted R-squared:  0.007359 
## F-statistic:  2.06 on 1 and 142 DF,  p-value: 0.1534
```

## Sensitivity analysis

If residual standard >3 AND hatvalue >2 times the average of hatvalues, run analysis with those cases deleted to test for sensitivity (from Habeck & Schultz 2015).

```
rs.Q1b.me<-rstandard (m.Q1b)
hat.Q1b.me<-hatvalues(m.Q1b)/mean(hatvalues(m.Q1b))
plot(hat.Q1b.me, rs.Q1b.me$resid, xlab="hat / average hat value", ylab= "standard residuals",xlim=c(0,2.5), ylim=c(-3,3), cex.lab=1.2)
abline (h=-3)
abline (h=3)
abline (v=(2))
```
