# Supplementary material for: Unravelling the gender productivity gap in science: a meta-analytical review
Source: R Soc Open Sci. 2019 Jun 12;6(6):181566. doi: 10.1098/rsos.181566 (PMC6599789; doi:10.1098/rsos.181566)
Supplement: Analyses code Q2.html [file rsos181566supp13.html]

Unravelling the gender productivity gap in science


# Unravelling the gender productivity gap in science

### Question 2 - Gender effect on success rate

#### Camila de Toledo Castanho

#### March 21, 2019

# Installing required packages

```
install.packages (c("metafor", "rmarkdown", "knitr")
```

# Loading required packages

```
library(knitr)
library(rmarkdown)
library (metafor)
```

```
## Loading required package: Matrix
```

```
## Loading 'metafor' package (version 2.0-0). For an overview 
## and introduction to the package please type: help(metafor).
```

# Loading data

```
Q2<-read.table("Q2.txt", header=TRUE, sep="\t")
str(Q2)
```

```
## 'data.frame':    43 obs. of  11 variables:
##  $ ID_article        : int  110 140 246 246 270 277 315 350 350 371 ...
##  $ ID_observation    : int  1 2 3 4 5 6 7 8 9 10 ...
##  $ Reference         : Factor w/ 27 levels "Abramo et al. 2016",..: 23 13 2 2 25 15 9 24 24 21 ...
##  $ Research_field    : Factor w/ 5 levels "Biological science",..: 1 2 2 2 1 3 2 3 3 2 ...
##  $ Productivity_proxy: Factor w/ 5 levels "evaluation_comitees",..: 2 5 2 2 5 2 5 2 2 5 ...
##  $ public_men        : int  265 965 340 90 563 537 193 25 51 226 ...
##  $ public_wom        : int  96 365 156 48 160 98 49 17 12 28 ...
##  $ rej_men           : int  416 1137 802 380 919 1983 589 856 1143 801 ...
##  $ rej_wom           : int  136 287 656 225 308 363 205 906 780 88 ...
##  $ N_men             : int  681 1855 1142 470 1482 2520 782 881 1194 1027 ...
##  $ N_wom             : int  232 652 812 273 468 461 254 923 792 116 ...
```

# Calculating effect sizes (yi) and variances (vi)

```
Q2<-escalc(measure="OR", ai=public_men, bi=rej_men,ci=public_wom, di=rej_wom, data=Q2)
str(Q2)
```

```
## Classes 'escalc' and 'data.frame':   43 obs. of  13 variables:
##  $ ID_article        : int  110 140 246 246 270 277 315 350 350 371 ...
##  $ ID_observation    : int  1 2 3 4 5 6 7 8 9 10 ...
##  $ Reference         : Factor w/ 27 levels "Abramo et al. 2016",..: 23 13 2 2 25 15 9 24 24 21 ...
##  $ Research_field    : Factor w/ 5 levels "Biological science",..: 1 2 2 2 1 3 2 3 3 2 ...
##  $ Productivity_proxy: Factor w/ 5 levels "evaluation_comitees",..: 2 5 2 2 5 2 5 2 2 5 ...
##  $ public_men        : int  265 965 340 90 563 537 193 25 51 226 ...
##  $ public_wom        : int  96 365 156 48 160 98 49 17 12 28 ...
##  $ rej_men           : int  416 1137 802 380 919 1983 589 856 1143 801 ...
##  $ rej_wom           : int  136 287 656 225 308 363 205 906 780 88 ...
##  $ N_men             : int  681 1855 1142 470 1482 2520 782 881 1194 1027 ...
##  $ N_wom             : int  232 652 812 273 468 461 254 923 792 116 ...
##  $ yi                : num  -0.103 -0.404 0.578 0.105 0.165 ...
##   ..- attr(*, "measure")= chr "OR"
##   ..- attr(*, "ni")= int  913 2754 1954 743 1950 2981 1036 1804 1986 1143 ...
##  $ vi                : num  0.02395 0.00814 0.01212 0.03902 0.01236 ...
##  - attr(*, "digits")= num 4
##  - attr(*, "yi.names")= chr "yi"
##  - attr(*, "vi.names")= chr "vi"
```

# Hierarchical mixed effect meta-analysis

```
m.Q2<-rma.mv(yi, vi, random=~1|ID_article/ID_observation,data=Q2)
m.Q2
```

```
## 
## Multivariate Meta-Analysis Model (k = 43; method: REML)
## 
## Variance Components: 
## 
##             estim    sqrt  nlvls  fixed                     factor
## sigma^2.1  0.1222  0.3495     27     no                 ID_article
## sigma^2.2  0.0672  0.2592     43     no  ID_article/ID_observation
## 
## Test for Heterogeneity: 
## Q(df = 42) = 666.9408, p-val < .0001
## 
## Model Results:
## 
## estimate      se    zval    pval   ci.lb   ci.ub     
##   0.3173  0.0900  3.5274  0.0004  0.1410  0.4936  ***
## 
## ---
## Signif. codes:  0 '***' 0.001 '**' 0.01 '*' 0.05 '.' 0.1 ' ' 1
```

```
forest (m.Q2, cex=0.4, slab=Q2$Reference, xlab="ln OR", mlab="Overall effect (43)")
text(-7.5,45, "Author(s) and Year", pos=4, font=2, cex=0.8)
text(8.5,45, "Ln OR [95% CI]", pos=2, font=2, cex=0.8)
```

# Heterogeneity

Heterogeneity I^2 for hierarchical models is not provided by metafor. We calculate total heterogeneity using the formulas provided by Nakagawa & Santos 2012. 1) Calculate sampling variance of the dataset (we use precision of effect size); 2) Use the variance components of the model associated with random factors (those summarized in the sigma2 structure components).

## Sampling variance of the dataset

```
Q2$wi <- 1/Q2$vi 
sv.mQ2 <- sum(Q2$wi*(length(Q2$wi)-1))/(sum(Q2$wi)^2-sum(Q2$wi^2))
sv.mQ2
```

```
## [1] 0.008160397
```

## Total heterogeneity

```
I2.total = (m.Q2$sigma2[1]+m.Q2$sigma2[2])/(m.Q2$sigma2[1]+m.Q2$sigma2[2] + sv.mQ2) * 100
I2.total
```

```
## [1] 95.86806
```

# Moderators

## Research field

### Significance of the moderator

This parameterization of the model is used to test the significance of the moderator.

```
m.Q2_field <- rma.mv(yi, vi, mods= ~Research_field,random=~1|ID_article/ID_observation, data=Q2)
m.Q2_field
```

```
## 
## Multivariate Meta-Analysis Model (k = 43; method: REML)
## 
## Variance Components: 
## 
##             estim    sqrt  nlvls  fixed                     factor
## sigma^2.1  0.1567  0.3959     27     no                 ID_article
## sigma^2.2  0.0563  0.2374     43     no  ID_article/ID_observation
## 
## Test for Residual Heterogeneity: 
## QE(df = 38) = 661.2354, p-val < .0001
## 
## Test of Moderators (coefficient(s) 2:5): 
## QM(df = 4) = 2.2113, p-val = 0.6970
## 
## Model Results:
## 
##                               estimate      se     zval    pval    ci.lb
## intrcpt                         0.3167  0.1787   1.7723  0.0763  -0.0335
## Research_fieldHealth            0.1020  0.2210   0.4617  0.6443  -0.3312
## Research_fieldMix              -0.2143  0.2810  -0.7628  0.4456  -0.7650
## Research_fieldSocial science   -0.2250  0.3601  -0.6249  0.5321  -0.9308
## Research_fieldTEMCP            -0.1968  0.3440  -0.5722  0.5672  -0.8710
##                                ci.ub   
## intrcpt                       0.6670  .
## Research_fieldHealth          0.5353   
## Research_fieldMix             0.3364   
## Research_fieldSocial science  0.4808   
## Research_fieldTEMCP           0.4774   
## 
## ---
## Signif. codes:  0 '***' 0.001 '**' 0.01 '*' 0.05 '.' 0.1 ' ' 1
```

### Estimation and significance of each level

This parameterization of the model is used to estimate the mean effect size of each level of the moderator and test which of them are different from zero.

```
m.Q2_field <- rma.mv(yi, vi, mods= ~ Research_field-1,random=~1|ID_article/ID_observation, data=Q2)
m.Q2_field
```

```
## 
## Multivariate Meta-Analysis Model (k = 43; method: REML)
## 
## Variance Components: 
## 
##             estim    sqrt  nlvls  fixed                     factor
## sigma^2.1  0.1567  0.3959     27     no                 ID_article
## sigma^2.2  0.0563  0.2374     43     no  ID_article/ID_observation
## 
## Test for Residual Heterogeneity: 
## QE(df = 38) = 661.2354, p-val < .0001
## 
## Test of Moderators (coefficient(s) 1:5): 
## QM(df = 5) = 13.5815, p-val = 0.0185
## 
## Model Results:
## 
##                                   estimate      se    zval    pval
## Research_fieldBiological science    0.3167  0.1787  1.7723  0.0763
## Research_fieldHealth                0.4188  0.1301  3.2196  0.0013
## Research_fieldMix                   0.1024  0.2168  0.4724  0.6366
## Research_fieldSocial science        0.0917  0.3126  0.2933  0.7693
## Research_fieldTEMCP                 0.1199  0.2939  0.4080  0.6833
##                                     ci.lb   ci.ub    
## Research_fieldBiological science  -0.0335  0.6670   .
## Research_fieldHealth               0.1638  0.6737  **
## Research_fieldMix                 -0.3225  0.5273    
## Research_fieldSocial science      -0.5211  0.7045    
## Research_fieldTEMCP               -0.4562  0.6960    
## 
## ---
## Signif. codes:  0 '***' 0.001 '**' 0.01 '*' 0.05 '.' 0.1 ' ' 1
```

### Change in the reference level

This parameterization is used to test if social science (as reference level) is significantly different from the other levels of the moderator.

```
m.Q2_field <- rma.mv(yi, vi, mods= ~ relevel (factor(Research_field), ref="Social science"),random=~1|ID_article/ID_observation,data=Q2)
m.Q2_field
```

```
## 
## Multivariate Meta-Analysis Model (k = 43; method: REML)
## 
## Variance Components: 
## 
##             estim    sqrt  nlvls  fixed                     factor
## sigma^2.1  0.1567  0.3959     27     no                 ID_article
## sigma^2.2  0.0563  0.2374     43     no  ID_article/ID_observation
## 
## Test for Residual Heterogeneity: 
## QE(df = 38) = 661.2354, p-val < .0001
## 
## Test of Moderators (coefficient(s) 2:5): 
## QM(df = 4) = 2.2113, p-val = 0.6970
## 
## Model Results:
## 
##                                                                            estimate
## intrcpt                                                                      0.0917
## relevel(factor(Research_field), ref = "Social science")Biological science    0.2250
## relevel(factor(Research_field), ref = "Social science")Health                0.3271
## relevel(factor(Research_field), ref = "Social science")Mix                   0.0107
## relevel(factor(Research_field), ref = "Social science")TEMCP                 0.0282
##                                                                                se
## intrcpt                                                                    0.3126
## relevel(factor(Research_field), ref = "Social science")Biological science  0.3601
## relevel(factor(Research_field), ref = "Social science")Health              0.3219
## relevel(factor(Research_field), ref = "Social science")Mix                 0.3145
## relevel(factor(Research_field), ref = "Social science")TEMCP               0.3637
##                                                                              zval
## intrcpt                                                                    0.2933
## relevel(factor(Research_field), ref = "Social science")Biological science  0.6249
## relevel(factor(Research_field), ref = "Social science")Health              1.0161
## relevel(factor(Research_field), ref = "Social science")Mix                 0.0340
## relevel(factor(Research_field), ref = "Social science")TEMCP               0.0776
##                                                                              pval
## intrcpt                                                                    0.7693
## relevel(factor(Research_field), ref = "Social science")Biological science  0.5321
## relevel(factor(Research_field), ref = "Social science")Health              0.3096
## relevel(factor(Research_field), ref = "Social science")Mix                 0.9728
## relevel(factor(Research_field), ref = "Social science")TEMCP               0.9382
##                                                                              ci.lb
## intrcpt                                                                    -0.5211
## relevel(factor(Research_field), ref = "Social science")Biological science  -0.4808
## relevel(factor(Research_field), ref = "Social science")Health              -0.3038
## relevel(factor(Research_field), ref = "Social science")Mix                 -0.6057
## relevel(factor(Research_field), ref = "Social science")TEMCP               -0.6846
##                                                                             ci.ub
## intrcpt                                                                    0.7045
## relevel(factor(Research_field), ref = "Social science")Biological science  0.9308
## relevel(factor(Research_field), ref = "Social science")Health              0.9580
## relevel(factor(Research_field), ref = "Social science")Mix                 0.6271
## relevel(factor(Research_field), ref = "Social science")TEMCP               0.7410
##                                                                             
## intrcpt                                                                     
## relevel(factor(Research_field), ref = "Social science")Biological science   
## relevel(factor(Research_field), ref = "Social science")Health               
## relevel(factor(Research_field), ref = "Social science")Mix                  
## relevel(factor(Research_field), ref = "Social science")TEMCP                
## 
## ---
## Signif. codes:  0 '***' 0.001 '**' 0.01 '*' 0.05 '.' 0.1 ' ' 1
```

## Productivity proxy

### Significance of the moderator

This parameterization of the model is used to test the significance of the moderator.

```
tapply(Q2$ID_observation, Q2$Productivity_proxy, length)
```

```
## evaluation_comitees              grants              others 
##                   2                  27                   3 
##            position        publications 
##                   3                   8
```

```
m.Q2_prod<-rma.mv(yi, vi, mods= ~ Productivity_proxy,random=~1|ID_article/ID_observation,data=Q2)
m.Q2_prod
```

```
## 
## Multivariate Meta-Analysis Model (k = 43; method: REML)
## 
## Variance Components: 
## 
##             estim    sqrt  nlvls  fixed                     factor
## sigma^2.1  0.0000  0.0000     27     no                 ID_article
## sigma^2.2  0.0925  0.3042     43     no  ID_article/ID_observation
## 
## Test for Residual Heterogeneity: 
## QE(df = 38) = 276.9898, p-val < .0001
## 
## Test of Moderators (coefficient(s) 2:5): 
## QM(df = 4) = 24.8038, p-val < .0001
## 
## Model Results:
## 
##                                 estimate      se     zval    pval    ci.lb
## intrcpt                           1.1546  0.2204   5.2376  <.0001   0.7225
## Productivity_proxygrants         -0.9853  0.2327  -4.2352  <.0001  -1.4413
## Productivity_proxyothers         -0.2578  0.3731  -0.6909  0.4896  -0.9890
## Productivity_proxyposition       -0.7864  0.2886  -2.7252  0.0064  -1.3520
## Productivity_proxypublications   -1.0751  0.2542  -4.2300  <.0001  -1.5732
##                                   ci.ub     
## intrcpt                          1.5866  ***
## Productivity_proxygrants        -0.5293  ***
## Productivity_proxyothers         0.4735     
## Productivity_proxyposition      -0.2208   **
## Productivity_proxypublications  -0.5770  ***
## 
## ---
## Signif. codes:  0 '***' 0.001 '**' 0.01 '*' 0.05 '.' 0.1 ' ' 1
```

### Estimation and significance of each level

This parameterization of the model is used to estimate the mean effect size of each level of the moderator and test which of them are different from zero.

```
m.Q2_prod<-rma.mv(yi, vi, mods= ~ Productivity_proxy-1,random=~1|ID_article/ID_observation,data=Q2)
m.Q2_prod
```

```
## 
## Multivariate Meta-Analysis Model (k = 43; method: REML)
## 
## Variance Components: 
## 
##             estim    sqrt  nlvls  fixed                     factor
## sigma^2.1  0.0000  0.0000     27     no                 ID_article
## sigma^2.2  0.0925  0.3042     43     no  ID_article/ID_observation
## 
## Test for Residual Heterogeneity: 
## QE(df = 38) = 276.9898, p-val < .0001
## 
## Test of Moderators (coefficient(s) 1:5): 
## QM(df = 5) = 45.7892, p-val < .0001
## 
## Model Results:
## 
##                                        estimate      se    zval    pval
## Productivity_proxyevaluation_comitees    1.1546  0.2204  5.2376  <.0001
## Productivity_proxygrants                 0.1693  0.0744  2.2752  0.0229
## Productivity_proxyothers                 0.8968  0.3010  2.9794  0.0029
## Productivity_proxyposition               0.3682  0.1862  1.9770  0.0480
## Productivity_proxypublications           0.0795  0.1265  0.6284  0.5298
##                                          ci.lb   ci.ub     
## Productivity_proxyevaluation_comitees   0.7225  1.5866  ***
## Productivity_proxygrants                0.0234  0.3151    *
## Productivity_proxyothers                0.3068  1.4868   **
## Productivity_proxyposition              0.0032  0.7332    *
## Productivity_proxypublications         -0.1685  0.3274     
## 
## ---
## Signif. codes:  0 '***' 0.001 '**' 0.01 '*' 0.05 '.' 0.1 ' ' 1
```

### Change in the reference level

This parameterization is used to test if published articles (as reference level) is significantly different from the other levels of the moderator.

```
m.Q2_prod<-rma.mv(yi, vi, mods= ~ relevel (factor(Productivity_proxy), ref="publications"),random=~1|ID_article/ID_observation,data=Q2)
m.Q2_prod
```

```
## 
## Multivariate Meta-Analysis Model (k = 43; method: REML)
## 
## Variance Components: 
## 
##             estim    sqrt  nlvls  fixed                     factor
## sigma^2.1  0.0000  0.0000     27     no                 ID_article
## sigma^2.2  0.0925  0.3042     43     no  ID_article/ID_observation
## 
## Test for Residual Heterogeneity: 
## QE(df = 38) = 276.9898, p-val < .0001
## 
## Test of Moderators (coefficient(s) 2:5): 
## QM(df = 4) = 24.8038, p-val < .0001
## 
## Model Results:
## 
##                                                                               estimate
## intrcpt                                                                         0.0795
## relevel(factor(Productivity_proxy), ref = "publications")evaluation_comitees    1.0751
## relevel(factor(Productivity_proxy), ref = "publications")grants                 0.0898
## relevel(factor(Productivity_proxy), ref = "publications")others                 0.8173
## relevel(factor(Productivity_proxy), ref = "publications")position               0.2887
##                                                                                   se
## intrcpt                                                                       0.1265
## relevel(factor(Productivity_proxy), ref = "publications")evaluation_comitees  0.2542
## relevel(factor(Productivity_proxy), ref = "publications")grants               0.1468
## relevel(factor(Productivity_proxy), ref = "publications")others               0.3265
## relevel(factor(Productivity_proxy), ref = "publications")position             0.2251
##                                                                                 zval
## intrcpt                                                                       0.6284
## relevel(factor(Productivity_proxy), ref = "publications")evaluation_comitees  4.2300
## relevel(factor(Productivity_proxy), ref = "publications")grants               0.6116
## relevel(factor(Productivity_proxy), ref = "publications")others               2.5032
## relevel(factor(Productivity_proxy), ref = "publications")position             1.2823
##                                                                                 pval
## intrcpt                                                                       0.5298
## relevel(factor(Productivity_proxy), ref = "publications")evaluation_comitees  <.0001
## relevel(factor(Productivity_proxy), ref = "publications")grants               0.5408
## relevel(factor(Productivity_proxy), ref = "publications")others               0.0123
## relevel(factor(Productivity_proxy), ref = "publications")position             0.1998
##                                                                                 ci.lb
## intrcpt                                                                       -0.1685
## relevel(factor(Productivity_proxy), ref = "publications")evaluation_comitees   0.5770
## relevel(factor(Productivity_proxy), ref = "publications")grants               -0.1979
## relevel(factor(Productivity_proxy), ref = "publications")others                0.1774
## relevel(factor(Productivity_proxy), ref = "publications")position             -0.1526
##                                                                                ci.ub
## intrcpt                                                                       0.3274
## relevel(factor(Productivity_proxy), ref = "publications")evaluation_comitees  1.5732
## relevel(factor(Productivity_proxy), ref = "publications")grants               0.3774
## relevel(factor(Productivity_proxy), ref = "publications")others               1.4573
## relevel(factor(Productivity_proxy), ref = "publications")position             0.7299
##                                                                                  
## intrcpt                                                                          
## relevel(factor(Productivity_proxy), ref = "publications")evaluation_comitees  ***
## relevel(factor(Productivity_proxy), ref = "publications")grants                  
## relevel(factor(Productivity_proxy), ref = "publications")others                 *
## relevel(factor(Productivity_proxy), ref = "publications")position                
## 
## ---
## Signif. codes:  0 '***' 0.001 '**' 0.01 '*' 0.05 '.' 0.1 ' ' 1
```

# Publication bias

## Egger’s regression

Egger’s regression using the meta-analytic residuals as the response variable and the precision as the moderator, as proposed by Nakagawa & Santos 2012 for hierarchical models. If the intercept of Egger’s regression is significantly different from zero, there is evidence of publication bias.

```
egger.Q2<-lm(residuals.rma(m.Q2)~Q2$vi)
summary(egger.Q2)
```

```
## 
## Call:
## lm(formula = residuals.rma(m.Q2) ~ Q2$vi)
## 
## Residuals:
##      Min       1Q   Median       3Q      Max 
## -1.08296 -0.27656 -0.07236  0.29504  1.51465 
## 
## Coefficients:
##             Estimate Std. Error t value Pr(>|t|)
## (Intercept) -0.08598    0.09085  -0.946    0.349
## Q2$vi        0.48203    0.45224   1.066    0.293
## 
## Residual standard error: 0.5125 on 41 degrees of freedom
## Multiple R-squared:  0.02696,    Adjusted R-squared:  0.003229 
## F-statistic: 1.136 on 1 and 41 DF,  p-value: 0.2927
```

## Sensitivity analysis

If residual standard >3 AND hatvalue >2 times the average of hatvalues, run analysis with those cases deleted to test for sensitivity (from Habeck & Schultz 2015).

```
rs.Q2.me<-rstandard (m.Q2)
hat.Q2.me<-hatvalues(m.Q2)/mean(hatvalues(m.Q2))
plot(hat.Q2.me, rs.Q2.me$resid, xlab="hat / average hat value", ylab= "standard residuals",xlim=c(0,2.5), ylim=c(-3,3), cex.lab=1.2)
abline (h=-3)
abline (h=3)
abline (v=(2))
```
