# Supplementary material for: Unravelling the gender productivity gap in science: a meta-analytical review
Source: R Soc Open Sci. 2019 Jun 12;6(6):181566. doi: 10.1098/rsos.181566 (PMC6599789; doi:10.1098/rsos.181566)
Supplement: Analyses code Q3.html [file rsos181566supp16.html]

Unravelling the gender productivity gap in science


# Unravelling the gender productivity gap in science

### Question 3 - Gender effect on scientific impact

#### Camila de Toledo Castanho

#### May 14, 2019

# Installing required packages

```
install.packages (c("metafor", "rmarkdown", "knitr")
```

# Loading required packages

```
library(knitr)
library(rmarkdown)
library (metafor)
```

```
## Loading required package: Matrix
```

```
## Loading 'metafor' package (version 2.0-0). For an overview 
## and introduction to the package please type: help(metafor).
```

# Loading data

```
Q3<-read.table("Q3.txt", header=TRUE, sep="\t", as.is=TRUE)
str(Q3)
```

```
## 'data.frame':    37 obs. of  14 variables:
##  $ ID_article    : int  69 69 82 82 145 145 145 145 145 145 ...
##  $ ID_observation: int  1 2 3 4 5 6 7 8 9 10 ...
##  $ Reference     : chr  "Addessi et al. 2012" "Addessi et al. 2012" "Slyder et al. 2011" "Slyder et al. 2011" ...
##  $ Self_citation : chr  "Included" "Included" "Included" "Included" ...
##  $ N_men         : int  72 88 210 134 20 20 20 20 20 20 ...
##  $ N_wom         : int  98 61 38 50 20 20 20 20 20 20 ...
##  $ Mean_men      : num  6.04 7.8 58.88 40.04 1.4 ...
##  $ Mean_wom      : num  5.28 7.15 76.26 39.56 1.5 ...
##  $ SD_men        : num  5.6 7.04 79.6 40.74 NA ...
##  $ SD_wom        : num  3.96 6.4 120.03 38.75 NA ...
##  $ d             : num  NA NA NA NA 0.0422 ...
##  $ var.d         : num  NA NA NA NA 0.1 ...
##  $ yi            : num  NA NA NA NA 0.041 -0.471 0.229 -0.502 0.092 -0.445 ...
##  $ vi            : num  NA NA NA NA 0.096 0.099 0.097 0.099 0.096 0.098 ...
```

# Calculating effect sizes (yi) and variances (vi)

For the five observations from Copenheaver et al. 2010, we calculated Hedges’d indirectly because the authors did not provide SD. We used a meta-calculator to estimate the Cohen’s d from the mean, the sample size and the statistics from inference tests. Then, the Cohen’s d was converted to Hedges’d by using the formula provided in Borestein et al. 2009 (page 27). That is the reason why the Hedges’d (yi) and its respective variance (vi) for these five observations are already calculated.

```
Q3<-escalc(measure="SMD", m1i=Mean_men, m2i=Mean_wom, sd1i=SD_men, sd2i=SD_wom, n1i=N_men, n2i=N_wom, data=Q3, vtype="UB", replace=FALSE)
head (Q3)
```

```
##   ID_article ID_observation               Reference Self_citation N_men
## 1         69              1     Addessi et al. 2012      Included    72
## 2         69              2     Addessi et al. 2012      Included    88
## 3         82              3      Slyder et al. 2011      Included   210
## 4         82              4      Slyder et al. 2011      Included   134
## 5        145              5 Copenheaver et al. 2010      Included    20
## 6        145              6 Copenheaver et al. 2010      Included    20
##   N_wom Mean_men Mean_wom SD_men SD_wom       d  var.d      yi     vi
## 1    98     6.04     5.28   5.60   3.96      NA     NA  0.1602 0.0242
## 2    61     7.80     7.15   7.04   6.40      NA     NA  0.0953 0.0278
## 3    38    58.88    76.26  79.60 120.03      NA     NA -0.1994 0.0312
## 4    50    40.04    39.56  40.74  38.75      NA     NA  0.0119 0.0275
## 5    20     1.40     1.50     NA     NA  0.0422 0.1000  0.0410 0.0960
## 6    20     0.60     1.20     NA     NA -0.4802 0.1027 -0.4710 0.0990
```

# Hierarchical mixed effect meta-analysis

```
m.Q3<-rma.mv(yi, vi, random=~1|ID_article/ID_observation,data=Q3)
m.Q3
```

```
## 
## Multivariate Meta-Analysis Model (k = 37; method: REML)
## 
## Variance Components: 
## 
##             estim    sqrt  nlvls  fixed                     factor
## sigma^2.1  0.0825  0.2872     16     no                 ID_article
## sigma^2.2  0.0000  0.0000     37     no  ID_article/ID_observation
## 
## Test for Heterogeneity: 
## Q(df = 36) = 101.5370, p-val < .0001
## 
## Model Results:
## 
## estimate      se    zval    pval    ci.lb   ci.ub   
##   0.1522  0.0818  1.8608  0.0628  -0.0081  0.3125  .
## 
## ---
## Signif. codes:  0 '***' 0.001 '**' 0.01 '*' 0.05 '.' 0.1 ' ' 1
```

```
forest (m.Q3, slab=Q3$Reference, cex=0.5, mlab= "Overall effect (37)", xlab= "Hedges'd")
text(-10,39, "Author(s) and Year", pos=4, font=2, cex=1)
text(12,39, "Hedges'd [95% CI]", pos=2, font=2, cex=1)
```

# Heterogeneity

Heterogeneity I^2 for hierarchical models is not provided by metafor. We calculate total heterogeneity using the formulas provided by Nakagawa & Santos 2012. 1) Calculate sampling variance of the dataset (we use precision of effect size); 2) Use the variance components of the model associated with random factors (those summarized in the sigma2 structure components).

## Sampling variance of the dataset

```
Q3$wi <- 1/Q3$vi 
sv.mQ3 <- sum(Q3$wi*(length(Q3$wi)-1))/(sum(Q3$wi)^2-sum(Q3$wi^2))
sv.mQ3
```

```
## [1] 0.01994164
```

## Total heterogenity

```
I2.total = (m.Q3$sigma2[1]+m.Q3$sigma2[2])/(m.Q3$sigma2[1]+m.Q3$sigma2[2] + sv.mQ3) * 100
I2.total
```

```
## [1] 80.53191
```

# Moderators

## Self-citation

### Significance of the moderator

This parameterization of the model is used to test the significance of the moderator.

```
m.Q3_cit<-rma.mv(yi, vi, mods= ~Self_citation,random=~1|ID_article/ID_observation,data=Q3)
m.Q3_cit
```

```
## 
## Multivariate Meta-Analysis Model (k = 37; method: REML)
## 
## Variance Components: 
## 
##             estim    sqrt  nlvls  fixed                     factor
## sigma^2.1  0.0945  0.3074     16     no                 ID_article
## sigma^2.2  0.0000  0.0000     37     no  ID_article/ID_observation
## 
## Test for Residual Heterogeneity: 
## QE(df = 35) = 98.8861, p-val < .0001
## 
## Test of Moderators (coefficient(s) 2): 
## QM(df = 1) = 0.1655, p-val = 0.6842
## 
## Model Results:
## 
##                        estimate      se    zval    pval    ci.lb   ci.ub
## intrcpt                  0.0757  0.2174  0.3481  0.7278  -0.3504  0.5017
## Self_citationIncluded    0.0964  0.2370  0.4068  0.6842  -0.3681  0.5609
##                         
## intrcpt                 
## Self_citationIncluded   
## 
## ---
## Signif. codes:  0 '***' 0.001 '**' 0.01 '*' 0.05 '.' 0.1 ' ' 1
```

### Estimation and significance of each level

This parameterization of the model is used to estimate the mean effect size of each level of the moderator and test which of them are different from zero.

```
m.Q3_cit<-rma.mv(yi, vi, mods= ~Self_citation-1, random=~1|ID_article/ID_observation,data=Q3)
m.Q3_cit
```

```
## 
## Multivariate Meta-Analysis Model (k = 37; method: REML)
## 
## Variance Components: 
## 
##             estim    sqrt  nlvls  fixed                     factor
## sigma^2.1  0.0945  0.3074     16     no                 ID_article
## sigma^2.2  0.0000  0.0000     37     no  ID_article/ID_observation
## 
## Test for Residual Heterogeneity: 
## QE(df = 35) = 98.8861, p-val < .0001
## 
## Test of Moderators (coefficient(s) 1:2): 
## QM(df = 2) = 3.4459, p-val = 0.1785
## 
## Model Results:
## 
##                        estimate      se    zval    pval    ci.lb   ci.ub
## Self_citationExcluded    0.0757  0.2174  0.3481  0.7278  -0.3504  0.5017
## Self_citationIncluded    0.1721  0.0944  1.8234  0.0682  -0.0129  0.3570
##                         
## Self_citationExcluded   
## Self_citationIncluded  .
## 
## ---
## Signif. codes:  0 '***' 0.001 '**' 0.01 '*' 0.05 '.' 0.1 ' ' 1
```

# Publication bias

## Egger’s regression

Egger’s regression using the meta-analytic residuals as the response variable and the precision as the moderator, as proposed by Nakagawa & Santos 2012 for hierarchical models. If the intercept of Egger’s regression is significantly different from zero, there is evidence of publication bias.

```
egger.Q3<-lm(residuals.rma(m.Q3)~Q3$vi)
summary(egger.Q3)
```

```
## 
## Call:
## lm(formula = residuals.rma(m.Q3) ~ Q3$vi)
## 
## Residuals:
##      Min       1Q   Median       3Q      Max 
## -1.32466 -0.24022 -0.10295  0.02488  2.70072 
## 
## Coefficients:
##             Estimate Std. Error t value Pr(>|t|)
## (Intercept) -0.04122    0.13857  -0.297    0.768
## Q3$vi        1.00606    0.60603   1.660    0.106
## 
## Residual standard error: 0.7296 on 35 degrees of freedom
## Multiple R-squared:  0.07299,    Adjusted R-squared:  0.0465 
## F-statistic: 2.756 on 1 and 35 DF,  p-value: 0.1058
```

## Sensitivity analysis

If residual standard >3 AND hatvalue >2 the times average of hatvalues, run analysis with those cases deleted to test for sensitivity (from Habeck & Schultz 2015).

```
rs.Q3.me<-rstandard (m.Q3)
hat.Q3.me<-hatvalues(m.Q3)/mean(hatvalues(m.Q3))
plot(hat.Q3.me, rs.Q3.me$resid, xlab="hat / average hat value", ylab= "standard residuals",xlim=c(0,3), ylim=c(-3,3), cex.lab=1.2)
abline (h=-3)
abline (h=3)
abline (v=(2))
```
