# Supplementary material for: Unravelling the gender productivity gap in science: a meta-analytical review
Source: R Soc Open Sci. 2019 Jun 12;6(6):181566. doi: 10.1098/rsos.181566 (PMC6599789; doi:10.1098/rsos.181566)
Supplement: Analyses code Q4.html [file rsos181566supp19.html]

Unravelling the gender productivity gap in science


# Unravelling the gender productivity gap in science

### Question 4 - Gender bias

#### Camila de Toledo Castanho

#### May 13, 2019

# Installing required packages

```
install.packages (c("metafor", "rmarkdown", "knitr")
```

# Loading required packages

```
library(knitr)
library(rmarkdown)
library (metafor)
```

```
## Loading required package: Matrix
```

```
## Loading 'metafor' package (version 2.0-0). For an overview 
## and introduction to the package please type: help(metafor).
```

# Loading data

```
Q4<-read.table("Q4.txt", header=TRUE, sep="\t")
str(Q4)
```

```
## 'data.frame':    18 obs. of  20 variables:
##  $ ID_article    : int  147 345 364 365 365 375 1185 1186 1187 1187 ...
##  $ ID_observation: int  1 2 3 4 5 6 7 8 9 10 ...
##  $ Reference     : Factor w/ 9 levels "Borsuk et al. 2009",..: 1 4 6 8 8 5 7 9 3 3 ...
##  $ Research_field: Factor w/ 9 levels "Behaviour","Biology, Psicology, Engeenering and Economis",..: 4 3 9 7 7 1 6 2 5 5 ...
##  $ Psychology    : Factor w/ 3 levels "mix","no","yes": 2 2 2 3 3 3 2 1 2 2 ...
##  $ success_men   : int  85 NA NA 45 49 16 335 111 16 11 ...
##  $ success_wom   : int  83 NA NA 29 36 26 172 228 9 14 ...
##  $ fail_men      : int  29 NA NA 17 12 14 NA NA 4 9 ...
##  $ fail_wom      : int  31 NA NA 36 8 9 NA NA 11 6 ...
##  $ N_men         : int  114 114 63 62 61 30 NA NA 20 20 ...
##  $ N_wom         : int  114 114 64 65 44 35 NA NA 20 20 ...
##  $ Mean_men      : num  NA 5.33 4.05 NA NA NA NA NA NA NA ...
##  $ Mean_wom      : num  NA 5.26 3.33 NA NA ...
##  $ SD_men        : num  NA 0.12 1.05 NA NA ...
##  $ SD_wom        : num  NA 0.12 1.08 NA NA ...
##  $ N_total       : int  237 228 127 127 105 65 507 339 40 40 ...
##  $ d             : num  NA NA NA NA NA ...
##  $ dvar          : num  NA NA NA NA NA NA 0.0053 0.0081 NA NA ...
##  $ yi            : num  NA NA NA NA NA ...
##  $ vi            : num  NA NA NA NA NA NA 0.0053 0.0081 NA NA ...
```

# Calculating effect sizes (yi) and variances (vi)

For the two observations from Reuben et al. 2014 and Williams & Ceci 2015, we calculated Hedges’d indirectly because the data provided are only based on two proportions. We used a meta-calculator to estimate the Cohen’s d from binary data (http://www.campbellcollaboration.org/escalc/html/EffectSizeCalculator-Home.php). Then, Cohen’s d was converted to Hedges’ d by using the formula provided in Borestein et al. 2009 pag 27. That is the reason why Hedges’ d (yi) and respective variance (vi) for the two observations are already calculated. For the remaining observations we have two kinds of data: based on means and binary data (2x2 tables).

## Hedges’d from means

```
Q4<-escalc(measure="SMD", m1i=Mean_men, m2i=Mean_wom, sd1i=SD_men, sd2i=SD_wom, n1i=N_men, n2i=N_wom, data=Q4, vtype="UB", replace=FALSE)
head (Q4)
```

```
##   ID_article ID_observation                       Reference Research_field
## 1        147              1              Borsuk et al. 2009        Ecology
## 2        345              2 Knobloch-Westerwick et al. 2013  Communication
## 3        364              3        Moss-Racusin et al. 2014        Science
## 4        365              4          Steinpreis et al. 1999     Phychology
## 5        365              5          Steinpreis et al. 1999     Phychology
## 6        375              6                      Lloyd 1990      Behaviour
##   Psychology success_men success_wom fail_men fail_wom N_men N_wom
## 1         no          85          83       29       31   114   114
## 2         no          NA          NA       NA       NA   114   114
## 3         no          NA          NA       NA       NA    63    64
## 4        yes          45          29       17       36    62    65
## 5        yes          49          36       12        8    61    44
## 6        yes          16          26       14        9    30    35
##   Mean_men Mean_wom SD_men SD_wom N_total  d dvar     yi     vi
## 1       NA       NA     NA     NA     237 NA   NA     NA     NA
## 2     5.33    5.260  0.120  0.120     228 NA   NA 0.5814 0.0183
## 3     4.05    3.325  1.055  1.085     127 NA   NA 0.6734 0.0333
## 4       NA       NA     NA     NA     127 NA   NA     NA     NA
## 5       NA       NA     NA     NA     105 NA   NA     NA     NA
## 6       NA       NA     NA     NA      65 NA   NA     NA     NA
```

## Hedges’d from binary data (2x2 tables)

```
Q4<-escalc(measure="OR2DN", ai=success_men, bi=fail_men,ci=success_wom, di=fail_wom,replace=FALSE,data=Q4)
head (Q4)
```

```
##   ID_article ID_observation                       Reference Research_field
## 1        147              1              Borsuk et al. 2009        Ecology
## 2        345              2 Knobloch-Westerwick et al. 2013  Communication
## 3        364              3        Moss-Racusin et al. 2014        Science
## 4        365              4          Steinpreis et al. 1999     Phychology
## 5        365              5          Steinpreis et al. 1999     Phychology
## 6        375              6                      Lloyd 1990      Behaviour
##   Psychology success_men success_wom fail_men fail_wom N_men N_wom
## 1         no          85          83       29       31   114   114
## 2         no          NA          NA       NA       NA   114   114
## 3         no          NA          NA       NA       NA    63    64
## 4        yes          45          29       17       36    62    65
## 5        yes          49          36       12        8    61    44
## 6        yes          16          26       14        9    30    35
##   Mean_men Mean_wom SD_men SD_wom N_total  d dvar      yi     vi
## 1       NA       NA     NA     NA     237 NA   NA  0.0548 0.0333
## 2     5.33    5.260  0.120  0.120     228 NA   NA  0.5814 0.0183
## 3     4.05    3.325  1.055  1.085     127 NA   NA  0.6734 0.0333
## 4       NA       NA     NA     NA     127 NA   NA  0.7210 0.0526
## 5       NA       NA     NA     NA     105 NA   NA -0.0589 0.0942
## 6       NA       NA     NA     NA      65 NA   NA -0.5620 0.1041
```

# Hierarchical mixed effect meta-analysis

```
m.Q4<-rma.mv(yi, vi, random=~1|ID_article/ID_observation,data=Q4)
m.Q4
```

```
## 
## Multivariate Meta-Analysis Model (k = 18; method: REML)
## 
## Variance Components: 
## 
##             estim    sqrt  nlvls  fixed                     factor
## sigma^2.1  0.2251  0.4744      9     no                 ID_article
## sigma^2.2  0.0275  0.1659     18     no  ID_article/ID_observation
## 
## Test for Heterogeneity: 
## Q(df = 17) = 214.3548, p-val < .0001
## 
## Model Results:
## 
## estimate      se    zval    pval    ci.lb   ci.ub   
##   0.1768  0.1761  1.0041  0.3153  -0.1684  0.5220   
## 
## ---
## Signif. codes:  0 '***' 0.001 '**' 0.01 '*' 0.05 '.' 0.1 ' ' 1
```

```
forest (m.Q4, slab=Q4$Reference, xlab="Hedges'd", ylim=c(-1,21), xlim=c(-10,10),mlab= "Overall effect (18)", cex=0.8)
text(-10,20, "Author(s) and Year", pos=4, font=2, cex=1.2)
text(10,20, "Hedges'd [95% CI]", pos=2, font=2, cex=1.2)
```

# Heterogeneity

Heterogeneity I^2 for hierarchical models is not provided by metafor. We calculate total heterogeneity using the formulas provided by Nakagawa & Santos 2012. 1) Calculate sampling variance of the dataset (we use precision of effect size); 2) Use the variance components of the model associated with random factors (those summarized in the sigma2 structure components).

## Sampling variance of the dataset

```
Q4$wi <- 1/Q4$vi
sv.mQ4 <- sum(Q4$wi*(length(Q4$wi)-1))/(sum(Q4$wi)^2-sum(Q4$wi^2))
sv.mQ4
```

```
## [1] 0.02489834
```

## Total heterogenity

```
I2.total = (m.Q4$sigma2[1]+m.Q4$sigma2[2])/(m.Q4$sigma2[1]+m.Q4$sigma2[2] + sv.mQ4) * 100
I2.total
```

```
## [1] 91.02849
```

# Moderators

## Research field: psychology or other fields

### Significance of the moderator

This parameterization of the model is used to test the significance of the moderator.

```
m.Q4_psy<-rma.mv(yi, vi, mods= ~ Psychology,random=~1|ID_article/ID_observation,data=Q4)
m.Q4_psy
```

```
## 
## Multivariate Meta-Analysis Model (k = 18; method: REML)
## 
## Variance Components: 
## 
##             estim    sqrt  nlvls  fixed                     factor
## sigma^2.1  0.0334  0.1827      9     no                 ID_article
## sigma^2.2  0.0338  0.1837     18     no  ID_article/ID_observation
## 
## Test for Residual Heterogeneity: 
## QE(df = 15) = 39.8338, p-val = 0.0005
## 
## Test of Moderators (coefficient(s) 2:3): 
## QM(df = 2) = 17.5739, p-val = 0.0002
## 
## Model Results:
## 
##                estimate      se     zval    pval    ci.lb    ci.ub     
## intrcpt         -0.7792  0.2743  -2.8405  0.0045  -1.3169  -0.2415   **
## Psychologyno     1.2704  0.3070   4.1386  <.0001   0.6688   1.8721  ***
## Psychologyyes    0.8827  0.3148   2.8045  0.0050   0.2658   1.4996   **
## 
## ---
## Signif. codes:  0 '***' 0.001 '**' 0.01 '*' 0.05 '.' 0.1 ' ' 1
```

### Estimation and significance of each level

This parameterization of the model is used to estimate the mean effect size of each level of the moderator and test which of them are different from zero.

```
m.Q4_psy<-rma.mv(yi, vi, mods= ~ Psychology-1,random=~1|ID_article/ID_observation,data=Q4)
m.Q4_psy
```

```
## 
## Multivariate Meta-Analysis Model (k = 18; method: REML)
## 
## Variance Components: 
## 
##             estim    sqrt  nlvls  fixed                     factor
## sigma^2.1  0.0334  0.1827      9     no                 ID_article
## sigma^2.2  0.0338  0.1837     18     no  ID_article/ID_observation
## 
## Test for Residual Heterogeneity: 
## QE(df = 15) = 39.8338, p-val = 0.0005
## 
## Test of Moderators (coefficient(s) 1:3): 
## QM(df = 3) = 21.2322, p-val < .0001
## 
## Model Results:
## 
##                estimate      se     zval    pval    ci.lb    ci.ub     
## Psychologymix   -0.7792  0.2743  -2.8405  0.0045  -1.3169  -0.2415   **
## Psychologyno     0.4912  0.1378   3.5656  0.0004   0.2212   0.7612  ***
## Psychologyyes    0.1035  0.1543   0.6707  0.5024  -0.1990   0.4060     
## 
## ---
## Signif. codes:  0 '***' 0.001 '**' 0.01 '*' 0.05 '.' 0.1 ' ' 1
```

# Publication bias

## Egger’s regression

Egger’s regression using the meta-analytic residuals as the response variable and the precision as the moderator, as proposed by Nakagawa & Santos 2012 for hierarchical models. If the intercept of Egger’s regression is significantly different from zero, there is evidence of publication bias.

```
egger.Q4<-lm(residuals.rma(m.Q4)~Q4$vi)
summary(egger.Q4)
```

```
## 
## Call:
## lm(formula = residuals.rma(m.Q4) ~ Q4$vi)
## 
## Residuals:
##      Min       1Q   Median       3Q      Max 
## -0.93445 -0.26347  0.01415  0.35810  0.79021 
## 
## Coefficients:
##             Estimate Std. Error t value Pr(>|t|)
## (Intercept) -0.02233    0.16226  -0.138    0.892
## Q4$vi        0.09031    2.25751   0.040    0.969
## 
## Residual standard error: 0.4845 on 16 degrees of freedom
## Multiple R-squared:  0.0001, Adjusted R-squared:  -0.06239 
## F-statistic: 0.0016 on 1 and 16 DF,  p-value: 0.9686
```

## Sensitivity analysis

If residual standard >3 AND hatvalue >2 the times average of hatvalues, run analysis with those cases deleted to test for sensitivity (from Habeck & Schultz 2015).

```
rs.Q4.me<-rstandard (m.Q4)
hat.Q4.me<-hatvalues(m.Q4)/mean(hatvalues(m.Q4))
plot(hat.Q4.me, rs.Q4.me$resid, xlab="hat / average hat value", ylab= "standard residuals",xlim=c(0,2.5), ylim=c(-6,6.5), cex.lab=1.2)
abline (h=-3)
abline (h=3)
abline (v=(2))
```
